# The quantum optics of media

**DOI:** 10.1098/rsta.2023.0339

**Published:** 2024-12-24

**Authors:** Stephen M. Barnett

**Affiliations:** ^1^ School of Physics and Astronomy, University of Glasgow, Glasgow G128QQ, UK

**Keywords:** quantized fields in media, optical forces, optical momenta

## Abstract

The quantum theory of light in real media requires attention to a number of physical features. Even in near-transparent dielectrics, we have to incorporate dispersion, losses and the effects of interfaces. Here, we review the quantization of light in a dielectric and see how this affects radiative processes and light propagation. In the second half of the paper, we turn to optical forces and momentum. There we show why there are two rival force densities in a medium and also why there *must be* two distinct optical momenta. In the process, this resolves the century-old Abraham–Minkowski dilemma.

This article is part of the theme issue ‘The quantum theory of light’.

## 1. Introduction

The early days of the development of quantum optics, certainly of theoretical studies, were dominated by discrete field modes, either encased in cavities or in a quantization box [1–5]. Single-mode quantum optics, in particular, has its origins in the development of the laser. Indeed the famous Jaynes–Cummings model appeared, initially, as an elementary maser model and was used to contrast quantum behaviour with that of a semiclassical theory; the latter treating the matter quantum mechanically but the field classically [6,7]. The discrete mode description has been highly fruitful, notably in the development of cavity quantum electrodynamics [8,9]. The contrast between fully quantum and semiclassical theories, moreover, was the underlying theme for much of quantum optics from the 1980s onwards [10–26]. Advancing technologies enabled the demonstration of a number of intrinsically non-classical effects including optical squeezing [27], photon anti-bunching [28], single-photon interference [29] and, perhaps most dramatically, the demonstration of the failure of local realism by means of the violation of Bell’s inequality [30–32].

The quantum optics of discrete modes has a drawback in that the *optics* is somehow missing. Optics is largely concerned with propagating fields and the interaction of these with macroscopic elements, such as lenses and mirrors, to produce images. A key advance was the introduction of continuum fields into the subject, adopting some of the elements of quantum field theory [33]. Early applications of this included the quantum optics of light propagation in optical fibres [34].

A key conceptual development was an adjustment in the concept of the photon. Early in the development of quantum optics, one would regularly come across the idea that a photon is an elementary excitation of a single mode. The introduction of the continuum description, however, called for a more general definition in which a photon is the elementary excitation of the electromagnetic field. The former were typically excitations of single-frequency cavity modes. The latter included, for example, single excitations in the form of optical pulses.

Releasing photons from optical cavities and allowing them to propagate introduces the challenge of determining how they interact with macroscopic objects. They will be slowed by passing through transparent (or partially transparent) media, possibly absorbed and, in turn, can exert forces and torques on the host object. Rodney Loudon made seminal contributions to the study of both the effects on the light of passing through media and also on the nature of the optical forces and torques exerted by the light on the medium. In this paper, we review the theory of light propagation through media and also the subtleties associated with optical forces. This includes explaining, for the first time to our knowledge, the reason for the existence of two rival force densities, and giving a simple physical explanation for the reason that there are *two* distinct optical momenta in a medium. Our aim is to explore and to explain the physics underlying the phenomena associated with light in optical media.

The paper is split, naturally, into two parts. In the first, we present the theory of light quantization in media and how quantum states of light propagate through, and are modified by, a medium. In the second, we turn our attention to optical forces and how the quantum theory of light in media has provided insight into the old problem of determining the momentum of light in a medium. The resolution is that *both* of the rival momenta are correct [35,36].

## 2. Light quantization in a medium

We shall be concerned in this paper with the properties of non-magnetic dielectric media in the absence of free charges and currents. The presence of such a dielectric will modify the electromagnetic field. The simplest way to understand this is to consider a uniform isotropic and unbounded medium and to write the total energy of the field in the form


(2.1) $$E=\int dV\frac{1}{2}\left(DE+\mu_{0}H^{2}\right),$$

where E, D and H are the electric, electric displacement and magnetic fields, respectively. For simplicity, let us approximate the dielectric constant ε by a real constant. The fact that, for a single photon of angular frequency ω, the normal-ordered expectation value of the electrical part of the energy density, $\langle :\int dV\frac{1}{2}DE:\rangle$, must be 1/2ℏω, tells us that the electric field operator is reduced by the factor $\sqrt{\varepsilon }$ compared to its free-space value.^1^ This is nothing more than the phenomenon, familiar from electrostatics, that the dielectric screens, and so reduces, the electric field strength [37]. More generally each of the field operators will be modified by the medium.

For some purposes, the above description, or simple variations of it suffices. Real media, however, exhibit both dispersion and also losses and these processes need to coexist to ensure causality, as embodied in the Kramers–Kronig relations [37,38]. To account for these features it is necessary to treat the medium itself quantum mechanically and, in particular, to quantize the medium polarization, which we can describe by a simple harmonic response or a set of such responses corresponding to the natural resonance frequencies of the medium. Coupling these produces a number of polariton modes; each frequency ω corresponds to a number of different polaritons. This is the familiar Hopfield model of the dielectric [39–42]. The photons of free space are replaced, in the medium, by polaritons which are excitations of the interacting electromagnetic field and the polarization.

The route to canonical quantization imposes two fixed relationships of the properties of the polariton branches. These are most naturally expressed in terms of the phase and group velocities ($v_{p}=\omega /k$ and $v_{g}=d\omega /dk$) for each of the branches. These relationships are [41,43,44]


(2.2) $$\begin{array}{lll} \sum_{i}\frac{v_{g}^{i}}{v_{p}^{i}} & = & 1,\\ \sum_{i}v_{g}^{i}v_{p}^{i} & = & c^{2}, \end{array}$$

where the summations run over all of the polariton modes and c is the speed of light in free space. These velocity sum rules are essential for consistency with the canonical commutation relations derived in the quantization procedure.

To account for losses owing to the inevitable absorption, we can couple the harmonic polarization to a reservoir, most readily described by a continuum of oscillators. This technique of coupling a system of interest to a bath of oscillators is a common procedure in quantum optics [4,17]. We then have the electric field coupled to a continuum of material modes; for each wave-vector 𝐤, which for the free field corresponds to a continuum of frequencies in the matter. We are then faced with the Hamiltonian [43,45]:


(2.3) $$\begin{array}{lll} \hat{H} & = & \sum_{\lambda }\{\int d^{3}k\;\hbar c\tilde{k}\;{\hat{a}}_{\lambda }^{†}(k){\hat{a}}_{\lambda }(k)+\int_{0}^{\infty }d\omega \hbar \omega {\hat{B}}_{\lambda }^{†}(k,\omega ){\hat{B}}_{\lambda }(k,\omega )\\ & & \;\;+\hbar \int_{0}^{\infty }d\omega \{\xi (\omega ){\hat{B}}_{\lambda }^{†}(k,\omega )[{\hat{a}}_{\lambda }(k)+{\hat{a}}_{\lambda }^{†}(-k)]+H.c.\}\}. \end{array}$$

Here, the electromagnetic field is described in terms of the continuum annihilation and creation operators, ${\hat{a}}_{\lambda }(𝐤$ and ${\hat{a}}_{\lambda }^{†}(𝐤)$, where $\left[{\hat{a}}_{\lambda }(𝐤),{\hat{a}}_{\lambda^{\prime }}^{†}({𝐤}^{\prime })]=\delta_{\lambda \lambda^{\prime }}\delta (𝐤-{𝐤}^{\prime }\right)$, and the damped medium polarization by the operators ${\hat{B}}_{\lambda }(𝐤,\omega )$ and ${\hat{B}}_{\lambda }^{†}(𝐤,\omega )$, where $\left[{\hat{B}}_{\lambda }(𝐤,\omega ),{\hat{B}}_{\lambda^{\prime }}^{†}({𝐤}^{\prime },\omega^{\prime })]=\delta_{\lambda \lambda^{\prime }}\delta (\omega -\omega^{\prime })\delta (𝐤-{𝐤}^{\prime }\right)$. The subscript λ denotes the two possible polarizations of the field and $c\tilde{k}$ is the frequency of the electromagnetic field shifted by its interaction with the medium; the function ξ(ω) represents the frequency-dependent coupling between the electromagnetic field and the material medium. Further details can be found in [43,46].

The Hamiltonian (equation (2.3)) is diagonalizable exactly [43,47] and results in the simple form


(2.4) $$\begin{array}{ccc} & \hat{H}=\sum_{\lambda }\int d^{3}k\int_{0}^{\infty }d\omega \;\hbar \omega \;{\hat{C}}_{\lambda }^{†}(𝐤,\omega ){\hat{C}}_{\lambda }(𝐤,\omega ), & \end{array}$$

where $\left[{\hat{C}}_{\lambda }(𝐤,\omega ),{\hat{C}}_{\lambda^{\prime }}^{†}({𝐤}^{\prime },\omega^{\prime })]=\delta_{\lambda \lambda^{\prime }}\delta (\omega -\omega^{\prime })\delta (𝐤-{𝐤}^{\prime }\right)$. The operators ${\hat{C}}_{\lambda }(𝐤,\omega )$ are the analogue of the polariton annihilation operators [39–42,48] but here, because of the damping, there is a continuum of possible wave-vectors for each frequency.

It is a straightforward matter to express the operators for the electromagnetic fields, and indeed for the material polarization, in terms of the dressed operators ${\hat{C}}_{\lambda }(𝐤,\omega )$ and to use these to verify that the field canonical commutation relations are indeed satisfied as required for self-consistency [43]. The techniques of field quantization in an absorbing medium are readily transferred to the study of simple amplifying media [49–51] and to magneto-dielectric media [52–54].

### (a) Medium-modified atomic transition rates

A quantum theory of light within a realistic medium makes it possible to examine a variety of elementary processes within the medium, specifically the emission and absorption of radiation. In particular, applying the Fermi golden rule to the evolution of a single excited atom within our medium gives a spontaneous emission rate that is the free-space rate multiplied by the real refractive index of the medium [55,56]. A more careful analysis suggests a modification of this decay rate because of local field corrections [55–58]. We note that such local field corrections have been the subject of extended studies in electromagnetic theory [37,59–62] but that they are not always required [61]. In a lossy medium, moreover, there exists a non-radiative decay rate associated with the interaction between the excited atom and the longitudinal electric field in the medium [56].

The spontaneous emission rate can be either enhanced or suppressed by the environment. Indeed the effect in general will be frequency dependent, with enhancement at some frequencies balanced by suppression at others. In general, the transverse decay rate (arising from coupling between the atom and the transverse electric field) at frequency ω, $\Gamma_{m}(\omega )$, is constrained by the sum rule [63,64]


(2.5) $$\begin{array}{ccc} & \int_{0}^{\infty }d\omega \frac{\Gamma_{m}(\omega )-\Gamma_{0}(\omega )}{\Gamma_{0}(\omega )}=0, & \end{array}$$

where $\Gamma_{0}(\omega )$ is the free-space decay rate. We note that for the simple result that the spontaneous emission is simply the product of the free-space decay rate and the (real part of) the refractive index η(ω) [55,56] this corresponds to the expression


(2.6) $$\begin{array}{ccc} & \int_{0}^{\infty }d\omega (\eta (\omega )-1)=0, & \end{array}$$

which is a well-known property of dielectrics and a consequence of the Kramers–Kronig relations [38,55,56].

The same ideas can be applied to determine the rates of absorption and stimulated emission by an atom [65,66]. Rather than pursue this, it is instructive to follow these authors and to use detailed balance, modifying the familiar relations between the A and B coefficients for an atom in free space [1]. Consider, for simplicity, a dilute gas of atoms or molecules, each with a single ground state and a single excited state, which we designate as state 1 and state 2, respectively, with $N_{1}$ and $N_{2}$ being the number of molecules in each of these two states. Detailed balance then requires that (on average)


(2.7) $$\begin{array}{rl} N_{2}A_{21}-N_{1}B_{12}\bar{W} & +N_{2}B_{21}\bar{W}=0\\ \Rightarrow \bar{W} & =\frac{A_{21}}{(N_{1}/N2)B_{12}-B_{21}}, \end{array}$$

where $A_{21}$ as the spontaneous emission rate, $B_{12}$ and $B_{21}$ are the Einstein B coefficients for absorption and emission and $\bar{W}$ is mean energy density per unit frequency interval [1]. We can determine the relationships between the A and B coefficients by comparing this expression for the energy density with that associated with the black body radiation field. Within a medium, however, this needs to be modified from that for free space:


(2.8) $$\begin{array}{ccc} & \bar{W}=\frac{\hbar \omega^{3}}{\pi^{2}c^{3}}\frac{1}{e^{\hbar \omega /k_{B}T}-1}\frac{\eta^{2}(\omega )c}{v_{g}(\omega )}. & \end{array}$$

Here, the final medium-dependent factor, which depends on the refractive index and also on the group velocity, arises from a combination of the modified field strength and the density of states within the medium [66]. Direct comparison of our two expressions for $\bar{W}$, together with the thermal equilibrium for our atoms or molecules, which requires that $N_{1}/N_{2}=e^{\hbar \omega /k_{B}T}$, leads to a simple expression for the medium-modified B coefficients. First, we find that $B_{12}=B_{21}$ and then


(2.9) $$\begin{array}{ccc} & \frac{A_{21}}{B}=\frac{\hbar \omega^{3}}{\pi^{2}c^{2}}\frac{\eta^{2}(\omega_{a})}{v_{g}(\omega_{a})}, & \end{array}$$

where $\omega_{a}$ is the atomic transition frequency. This, together with the fact that the spontaneous emission rate in the medium and its value in free space are related by $A_{21}=\eta (\omega_{a})A_{21}^{0}$, means that the B coefficient is modified in comparison with its free-space value by


(2.10) $$\begin{array}{ccc} & B=\frac{v_{g}(\omega_{a})}{c\eta (\omega_{a})}B^{0}=\frac{B^{0}}{\eta (\omega_{a})\eta_{g}(\omega_{a})}, & \end{array}$$

where $\eta_{g}(\omega_{a})$ is the group index of the medium. It is interesting to note that for most frequencies in most media, the effect of the medium is to enhance the spontaneous emission rate while suppressing the absorption and stimulated emission rates.

### (b) Light propagation

As is often the case in physics, the essential features of a physical phenomenon are most readily appreciated by reference to a simplified model system. This is true also for the propagation of light in and into a linear dielectric medium. In texts on electromagnetism, the transmission and reflection at an interface are often simplified by approximating the light by a plane wave [37], and this can be further simplified by restricting the plane wave to propagate in the direction normal to any interfaces. There are certainly texts that consider the issues of transmission and reflection in greater depth and generality [67], but this is the situation in which we specialize here, so that the system reduces to a single spatial dimension and, within this, we need to consider only a single polarization. We empasize that this is not a fundamental restriction on the theory, but merely a simplification to reduce the complexity of our presentation.

Within these simplifications, we can write vector potential in our dispersive and lossy dielectric medium in the form [68,69]


(2.11) $$\begin{array}{ccc} & \hat{A}(x,t)=\int_{0}^{\infty }d\omega \left(\frac{\hbar \eta (\omega )}{4\pi \varepsilon_{0}Sc|n(\omega ){|}^{2}}\right)\{[{\hat{c}}_{+}(x,\omega )+{\hat{c}}_{-}(x,\omega )]e^{-i\omega t}+H.c.\}, & \end{array}$$

where S is the quantization area normal to the direction of propagation [33] and n(ω) is the complex refractive index, the real part of which is η(ω). The subscripts + and − correspond to propagation to the right and left, respectively, and satisfy the propagation equations [43,45,68]


(2.12) $$\frac{\partial {\hat{c}}_{\pm }(x,\omega )}{\partial x}=\pm i\frac{n(\omega )\omega }{c}{\hat{c}}_{\pm }(x,\omega )\pm {\left(\frac{2\kappa (\omega )\omega }{c}\right)}^{1/2}\hat{f}(x,\omega ),$$

where κ(ω) is the absorption coefficient (n=η+iκ) and $\hat{f}(x,\omega )$ is a Langevin noise operator:


(2.13) $$\begin{array}{ccc} & [\hat{f}(x,\omega ),{\hat{f}}^{†}(x^{\prime },\omega^{\prime })]=\delta (x-x^{\prime })\delta (\omega -\omega^{\prime }). & \end{array}$$

The evolution of the operators ${\hat{c}}_{+}$ and ${\hat{c}}_{-}$ is reminiscent of that for the temporal evolution of an annihilation operator for a discrete field mode coupled to a reservoir [4,17]. It is interesting to note that the operators corresponding to the right- and left-hand propagating fields are indirectly coupled by virtue of interacting with the same absorbing medium, and it follows that the commutation relations for these operators have the form [69]


(2.14) $$\begin{array}{rl} \left[{\hat{c}}_{+}(x,\omega ),{\hat{c}}_{+}^{†}(x^{\prime },\omega^{\prime })\right] & =[{\hat{c}}_{-}(x^{\prime },\omega^{\prime }),{\hat{c}}_{-}^{†}(x,\omega )]\\ & =\delta (\omega -\omega^{\prime })\exp\{[i\omega \eta (\omega )(x-x^{\prime })-\omega \kappa (\omega )|x-x^{\prime }|]/c\}\\ \left[{\hat{c}}_{+}(x,\omega ),{\hat{c}}_{-}^{†}(x^{\prime },\omega^{\prime })\right] & =[{\hat{c}}_{-}(x^{\prime },\omega^{\prime }),{\hat{c}}_{+}^{†}(x,\omega )]\\ & =\delta (\omega -\omega^{\prime })H(x-x^{\prime })\frac{2\kappa (\omega )}{\eta (\omega )}\sin\left(\frac{\omega \eta (\omega )(x-x^{\prime })}{c}\right)\exp\left(-\frac{\omega \kappa (\omega )(x-x^{\prime })}{c}\right), \end{array}$$

where $H(x-x^{\prime })$ is the Heaviside step function [70]. That the commutator for the right- and left-hand propagating fields is proportional to the absorption coefficient is evidence that the non-commutativity arises from coupling to the Langevin force associated with the absorption.

The theory is completed by using the familiar electromagnetic boundary conditions to couple the fields inside the medium to those outside. The result, for a slab of material with a finite thickness, is a scattering between incoming light from the right- and left-hand regions to produce outgoing fields together with losses inside the medium [69]. The effect of such devices on input fields was explored in detail in [71]. There are unexpected features in this, for example, there exist configurations in which a pair of incident photons are either both absorbed or both survive in an apparent nonlinear absorption, despite the fact that the medium is purely linear. Recent technological advances have renewed interest in such devices [72–77]. There are also non-local effects that can occur between entangled photons, each propagating through its own dispersive and absorbing or aberrating medium [78–80].

## 3. Optical forces and momenta

The possibility that light can exert forces dates back at least to the work of Kepler, who suggested that light from the sun was responsible for the observed tails of comets. In 1625, he wrote [81]: ‘… the train or beard is an effluvium from the head, expelled through the rays of sun into the opposed zone, and in its continued effusion the head is finally exhausted and consumed, so that the tail represents the death of the head.’ More than 200 years later Maxwell calculated the radiation pressure owing to sunlight on the surface of the earth [82]: ‘Thus if in strong sunlight the energy of the light which falls on one square foot is 83.4 foot pounds per second, the mean energy in one cubic foot of sunlight is about 0.0000000882 of a foot pound, and the mean pressure on a square foot is about 0.0000000882 of a pound weight.’ This equates to approximately 4μPa in modern units, which is the currently accepted value.

The modern experimental study of optical forces can be traced back to the work of Ashkin [83,84] and led, in the following years, to the development of optical tweezers, neutral traps and numerous related effects and devices [85]. This is not the place to review all of this work, and we shall consider here just the off-resonant forces exerted by light on a macroscopic object, in the spirit of Maxwell’s study. Employing quantum theory for this purpose brings with it the conceptual advantage that we can calculate the, in practice tiny, forces exerted by a single photon. This makes it possible to determine the forces per photon, and also to restrict attention to situations (by post-selection) in which the photon propagates without the complication of reflections. In our study of single-photon forces, we shall encounter a paradox or dilemma, which is over a century old: the puzzle of the correct form of the momentum carried by a photon on propagation through a dielectric medium [36,86–88]. There are two principal contenders, the Abraham and the Minkowski momenta which are, respectively, less or greater than the free-space momentum by a factor of the medium refractive index.

### (a) A tale of two forces …

The application of electromagnetic forces to the problem of optical momentum starts with a further problem: there are two candidate forces, or more precisely force densities, to be considered. The first of these arises from a microscopic model in which the medium is considered as a collection of bound charges [89,90]:


(3.1) $$f^{c}=-(\nabla \cdot P)E+\dot{P}\times B,$$

where 𝐏 is the medium polarization. The second is a consequence of treating the medium as a collection of neutral point dipoles [91–93]:


(3.2) $$f^{d}=(P\cdot \nabla )E+\dot{P}\times B.$$

We see that the electric parts of these force densities are different and may even point in different directions! Both of these have been used to calculate forces acting on a variety of material objects [89,90,94–96]. Force densities are not measured, however, forces are and it is straightforward to show that the total force acting on an isolated body is the same whichever force density is used [93]. The proof is simple enough that it is worth repeating here. Consider the difference in the i-component of the total force acting on an isolated dielectric in vacuum:


(3.3) $$\begin{array}{rl} F_{i}^{d}-F_{i}^{c} & =\int dV(P\cdot \nabla +\nabla \cdot P)E_{i}\\ & =\sum_{j}\int dV\nabla_{j}(P_{j}E_{i})\\ & =\int dS_{j}P_{j}E_{i}, \end{array}$$

where the surface integral encloses the entire object. However, such a surface can be taken to be outside the object where the polarization is zero, and hence the two forces are *equal*.

What has not been explained before, at least to the best of our knowledge not in print, is the reason for the existence of the two rival force densities. We present this here. To start at the beginning we return to the Lorentz force law in the form


(3.4) $$\begin{array}{ccc} & {𝐟}^{L}=\rho 𝐄+𝐉\times 𝐁. & \end{array}$$

(It is interesting to note that the force law is usually presented in the form of a force acting on a point charge, but Lorentz published a force as the integral of a force density [97].) The difference between ${𝐟}^{d}$ and ${𝐟}^{c}$ appears only in the electrical part and so we focus our attention on this.

**Figure 1.**
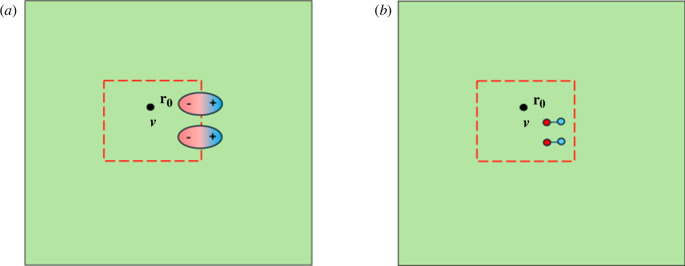
The effect of an (inhomogeneous) electric field on a region within a dielectric. (*a*) For a medium considered as a collection of bound charges and (*b*) for a medium comprised point dipoles.


In figure 1, we depict a small region within a dielectric and the effect of an imposed, macroscopic, electric field on this. In (a) the medium is considered to be formed from bound charges that can move independently and so can be displaced into or out of the region, so that our element of the dielectric need not be electrically neutral. In (b) the medium is composed of (point) dipoles. These can be oriented and stretched by the electric field but, in the process, our element remains electrically neutral. We can determine the total force acting on the element in each of these cases [93]. In doing so we consider the element to be sufficiently small so that the macroscopic electric field varies only very little across the element. This allows us to approximate the field by

(3.5) $$E(r)\approx E(r_{0})+{[(r-r_{0})\cdot \nabla ]E|}_{r_{0}},$$

where ${𝐫}_{0}$ is a point within the volume element. Hence the electric force acting on the element is


(3.6) $$\begin{array}{rl} f^{L} & =\int dV\;>\rho (r)E(r)\\ & \approx E(r_{0})\int dV\;>\rho (r)+\left\{\int dV\rho (r)[(r-r_{0})\cdot \nabla ]\right\}E(r_{0}). \end{array}$$

For the model based on bound charges, the first term is non-zero and leads to the force density −(∇⋅P)E and the second term can be neglected in the small volume limit. To see this, we note that, from Maxwell’s equations, ρ=−∇⋅P and then treating 𝐏 as the macroscopic polarization yields the desired result. For the dipole model, however, the volume is strictly neutral and the first term is identically zero. In this case, it is the second term that leads to the force density (P⋅∇)E. Here, we can adopt an alternative form for the polarization as the density of the dipoles, or the displacement of the each of the charges from a fixed point, which for our small volume is simply $\rho (𝐫)()𝐫-{𝐫}_{0}$. Thus, *both* force densities, ${𝐟}^{c}$ and ${𝐟}^{d}$, derive from the same Lorentz law but, when transformed into a force, they correspond to integration within the medium over slightly (but importantly) different volumes.

It is these fundamentally different volume elements that lead to the necessity for the existence of two possible force densities. If the volume element is chosen in such a way that charges can enter or leave it, then ${𝐟}^{c}$ will be the appropriate force density. In this case, the volume would include only part of the stretched dipoles near its surface as in figure 1*a*. A non-uniform electric field then produces a net charge in the volume [37]. If, however, the volume element retains fully all the dipoles enclosed then it is ${𝐟}^{d}$ that should be used. We follow this line of reasoning to address what might happen with an external field acting on a fluid. The field can induce motion in the fluid, but the signature of this will (probably) be the motion of the (neutral) molecules and, for this reason, it should be ${𝐟}^{c}$ that would reproduce the observed induced flow within the fluid.

### (b) … and of two momenta

The mystery of the form of the momentum of light in a medium has been debated for over a hundred years. The leading (and only serious, or at least established) contenders are the forms proposed by Minkowski in 1908 [98] and by Abraham in 1909 [99–101]. When expressed as a momentum density, the Abraham and Minkowski forms become $𝐄\times 𝐇/c^{2}$ and 𝐃×𝐁, respectively. In free space, these quantities are identical and for a single photon reduce to a magnitude ℏω/c. In a transparent medium, however, they give very different momenta. To see this, we recall that the electromagnetic energy density in the medium can be written in the form 1/2(D⋅E+B⋅H). For our single photon, each of the electric and magnetic parts of this contribute 1/2ℏω to the total energy and hence the fields scale as $𝐄∼{𝐄}_{0}/\sqrt{\varepsilon }$, $𝐃∼{𝐃}_{0}\sqrt{\varepsilon }$, $𝐇∼{𝐇}_{0}/\sqrt{\mu }$ and $𝐁∼{𝐁}_{0}\sqrt{\mu }$, where the fields written with a subscript 0 are the free-space forms [36]. From these, we can infer that the Abraham momentum is *less* than the free-space form by the factor $\sqrt{\varepsilon \mu }$, which is the refractive index. The Minkowski momentum, however, is *greater* than the free-space value by the same factor. The difference between these two momenta is usually not small; even for glass it is in excess of a factor of two.

There are strong physical arguments that can be made in favour of both candidate momenta. We have summarized these in an earlier publication [36], but it is worth pausing to give a brief restatement of two of these. We start with an argument in support of the Abraham momentum [102]. The physical principle in this case is the idea that the centre of mass (or more precisely mass-energy) of an isolated system continues in uniform motion [103]. Consider a single photon incident on a block of transparent material. Let the photon pass into the material and then out of the other side without undergoing any reflection. The fact that the photon moves more slowly in the glass than in free space means that after the photon has left, the block is shifted in the direction of propagation by an amount Δz where


(3.7) $$\begin{array}{ccc} & \Delta zMc^{2}=(n_{g}-1)L\hbar \omega \;\Rightarrow \;\Delta z=(n_{g}-1)L\frac{\hbar \omega }{Mc^{2}}, & \end{array}$$

where $n_{g}$ is the group refractive index of the block and M and L are the mass and thickness of the block, respectively. It follows that the block must have been in motion while the photon was passing through it and conservation of momentum ensures that the remaining momentum must have been carried by the photon, from which we can determine


(3.8) $$\begin{array}{ccc} & p_{\mathrm{Abr}}=\frac{\hbar \omega }{cn_{g}}, & \end{array}$$

so the photon momentum is reduced compared to its free-space value by the group index.

The simplest argument in favour of the Minkowski momentum follows from de Broglie’s observation that the momentum of a particle is h/λ and the wavelength in the block is decreased by the phase index of refraction, $n_{p}$, so that


(3.9) $$\begin{array}{ccc} & p_{\mathrm{Min}}=\frac{h}{\lambda }=\frac{\hbar \omega n_{p}}{c}. & \end{array}$$

We can test this idea by reference to single-slit diffraction by making the following argument [104]. We can estimate the width of the central diffraction peak using the uncertainty principle. If z is the direction of propagation and the slit is in the x direction, then the width is given by the angular spread


(3.10) $$\begin{array}{ccc} & \theta \approx \frac{\Delta p_{x}}{p_{z}}\approx \frac{\hbar }{p_{z}\Delta x}. & \end{array}$$

If the region between the slit and the imaging screen is filled with a dielectric with phase index $n_{p}$ then we find that θ is reduced by the factor $n_{p}$ [105]. It necessarily follows that


(3.11) $$\begin{array}{ccc} & p_{z}=\frac{\hbar \omega n_{p}}{c}, & \end{array}$$

which is the Minkowski result.

Thus, there are strong physical grounds for supporting both momenta and it is not surprising that there have been a number of experimental tests supporting either one momentum or the other [106–112]. Yet the resulting situation is not at all clear [87,88], for although most of these support the Minkowski form, others indicate that it should be the Abraham momentum that is correct. Moreover, some of these experimental results appear to be more subtle than initially thought [91,113].

It was Loudon’s insight that the problem might be addressed directly using optical forces to evaluate, through Newton’s second law of motion, the rate of change of momentum and hence to determine which momentum might be correct [89,93,94,114]. This needs to be done with some care as even the force exerted on a single atom is not without its subtleties [115]. This idea has been applied to analyse a number of different physical phenomena in which either optical forces or torques act [54,89,90,93–96,114,116,117]. In particular, a re-analysis, using optical forces, of the momentum transfer to a transparent block, in the argument described above, gives a momentum transfer in agreement with the Abraham expression (as it must) [118]. Photon drag experiments [119] detect long wavelength radiation by inducing a current in a suitably doped semiconductor, and the measurements of this effect are consistent with the Minkowski momentum. Once again, a calculation based on the optical force gives a result in full agreement with this [118].

It seems that we are no further forward, but the resolution of the dilemma we find ourselves in is that *both* momenta are correct [35,36]. The question we have to answer is why should light in a medium have two different and distinct momenta? We have addressed the technicalities of this in some detail elsewhere, as well as discussing previous work on the topic [35,36]. Here, we take the opportunity to present a simpler, if less rigorous, but hopefully physically appealing treatment.

We start by writing down a Hamiltonian for a dipole atom coupled to the surrounding electromagnetic field. It is helpful, although not strictly necessary, to treat this quantum mechanically. Hence we write (as a first guess) the electric-dipole Hamiltonian in the form [20]


(3.12) $$\begin{array}{ccc} & H=\frac{p^{2}}{2M}-𝐝\cdot 𝐄+H_{\mathrm{at}}+H_{\mathrm{rad}}, & \end{array}$$

where 𝐩 is the momentum operator for the atom, M is its mass, 𝐝 is the electric-dipole operator and $H_{\mathrm{at}}$ and $H_{\mathrm{rad}}$ are, respectively, the Hamiltonians for the internal degrees of freedom of the atom and for the electromagnetic field. The first term should be the kinetic energy associated with the centre of mass of the atom. We say ‘should be’ because, even in this non-relativistic analysis, relativity has a role to play. The first issue is that our atom should couple to the electric field in its *own rest frame* and to this end we should replace the electric field 𝐄 by 𝐄+𝐯×𝐁, but we have not yet established what the velocity of the atom is. A natural first try is to put 𝐯=𝐩/M, which gives


(3.13) $$\begin{array}{ccc} & H=\frac{p^{2}}{2M}-𝐝\cdot \left(𝐄+\frac{𝐩}{M}\times 𝐁\right)+H_{\mathrm{at}}+H_{\mathrm{rad}}. & \end{array}$$

We can use this to calculate the velocity of the atom through the Heisenberg equation of motion (we could equally well treat the problem classically and use Hamilton’s equations):


(3.14) $$\begin{array}{rl} \frac{d}{dt}r & =\frac{i}{\hbar }[H,r]\\ & =\frac{1}{M}(p+d\times B)\\ \Rightarrow M\dot{r} & =p+d\times B. \end{array}$$

It is clear, now, that there are *two* momenta appearing, a canonical momentum 𝐩 and a kinetic momentum Md𝐫/dt. The presence of two momenta is a direct consequence of the existence of a velocity-dependent interaction and is ubiquitous in quantum electrodynamics and, indeed, in its classical counterpart. Finally, the first term in our Hamiltonian should be the kinetic energy of the centre of mass, which is $1/2M{\dot{r}}^{2}$ and so we find the final form:


(3.15) $$\begin{array}{ccc} & \hat{H}=\frac{1}{2M}(𝐩+𝐝\times 𝐁{)}^{2}-𝐝\cdot 𝐄+H_{\mathrm{at}}+H_{\mathrm{rad}}, & \end{array}$$

which is the form arrived at more rigorously through the multipolar expansion [120–122]. Of course, this treatment is not a derivation and, indeed, the Hamiltonians (equations (3.12), (3.13) and (3.15)) are clearly not equivalent, but we hope that the physical origin of the two momenta, arising as a consequence of relativity, is clear.

The important feature of this, for our purposes, is the relationship between the canonical and the kinetic momenta encapsulated in equation (3.14). We can connect this property of a single dipole with our two optical momenta by considering the effect of an off-resonant light pulse on a single atom [115]. Here, however, we seek to understand the two momenta in the context of propagation in a macroscopic medium and we address this by considering the medium to be composed of many dipoles, so that the total kinetic and canonical momenta are related by


(3.16) $$\begin{array}{rl} M_{tot}{\dot{r}}_{CoM} & =\sum_{i}M{\dot{r}}_{i}\\ & =p_{tot}+\int dVP\times B, \end{array}$$

where ${\dot{𝐫}}_{\mathrm{CoM}}$ is the velocity of the centre of mass of the dielectric body, with mass $M_{\mathrm{tot}}$. The total canonical momentum is ${𝐩}_{\mathrm{tot}}$ and 𝐏 is the medium polarization. If we add to the kinetic momentum of the dielectric the Abraham momentum of the electromagnetic field then we recover a *single* globally conserved total momentum


(3.17) $$\begin{array}{ccc} & M_{\mathrm{tot}}{\dot{𝐫}}_{\mathrm{CoM}}+\frac{1}{c^{2}}\int dV𝐄\times 𝐇={𝐩}_{\mathrm{tot}}+\int dV𝐃\times 𝐁. & \end{array}$$

On the left-hand side of this equality, the momentum is divided between the matter and the field by kinetic quantities and on the right-hand side by canonical ones. It is clear that the two rival momenta are nothing more than the kinetic and the canonical momenta of the field [35,36]. This distinction is helpful, moreover, in suggesting which of the two momenta may be associated with any given phenomenon. Broadly speaking, the kinetic momentum is associated with particle-like properties of light while the canonical appears in wave-like phenomena, but there further subtleties [35].

There is one further insight that we can offer into the existence of a pair of distinct momenta for light in a medium. The distinction between the kinetic and canonical forms of the momentum, expressed in equation (3.17) comes down to whether we associate the component ∫dV𝐏×𝐁 with the matter or with the electromagnetic field. It is clearly part of the total momentum but contains *both* a matter quantity, 𝐏, and a field one, 𝐁. The kinetic and canonical decompositions of the total momentum correspond to including this mixed part with the momentum of, respectively, the matter or the field.

## 4. Conclusion

The presence of material media can have a profound effect on the quantized electromagnetic field. In the simplest situations, we have collective excitations of the coupled light-matter system in the form of polaritons [39–41]. Incorporating absorption losses in the medium requires the introduction of a reservoir coupled to the material polarization. The Hamiltonian of modelling this more complicated system can be diagonalized exactly [43] and this leads to a simple way to treat optical processes occurring in or near material media, including radiative processes and the propagation of quantum states of light.

It has long been known that optical fields can exert forces on material bodies. These forces underly optical traps, optical tweezers and the effects of radiation pressure [85]. There are two rival forms for the optical force density but these produce the same net or total forces [93]. We have explained the reason for the existence of the two optical force densities and determined that the force density based on microscopic dipoles is the one that should be used when considering the optical forces on fluids.

The optical forces provide the means by which to calculate momentum transfer from light to matter [118]. This allows us to determine that the two rival momenta, owing to Minkowski and to Abraham, are *both* correct and are manifest in distinct situations [35,36]. We have given a simple physical explanation for the existence of the two optical momenta as the electromagnetic canonical and kinetic momenta.

## Data Availability

This article has no additional data.

## Footnotes

^1^
The other half of the photon energy derives from the magnetic energy.
